# Adiponectin: a pleiotropic hormone with multifaceted roles

**DOI:** 10.14341/probl12827

**Published:** 2021-12-30

**Authors:** S. S. Shklyaev, G. A. Melnichenko, N. N. Volevodz, N. A. Falaleeva, S. A. Ivanov, A. D. Kaprin, N. G. Mokrysheva

**Affiliations:** National Research Center for Endocrinology of the Ministry of Health of the Russian Federation; A. Tsyb Medical Radiological Research Center — Branch of the National Medical Research Radiological Center of the Ministry of Health of the Russian Federation; National Research Center for Endocrinology of the Ministry of Health of the Russian Federatio; National Research Center for Endocrinology of the Ministry of Health of the Russian Federatio; A. Tsyb Medical Radiological Research Center — Branch of the National Medical Research Radiological Center of the Ministry of Health of the Russian Federation; A. Tsyb Medical Radiological Research Center — Branch of the National Medical Research Radiological Center of the Ministry of Health of the Russian Federation; A. Tsyb Medical Radiological Research Center — Branch of the National Medical Research Radiological Center of the Ministry of Health of the Russian Federation; National Research Center for Endocrinology of the Ministry of Health of the Russian Federation

**Keywords:** adipose tissue, adiponectin, AdipoR1, AdipoR2, T-cadherin, calreticulin, metabolic inflammation, ‘adiponectin paradox’

## Abstract

Adipose tissue mostly composed of different types of fat is one of the largest endocrine organs in the body playing multiple intricate roles including but not limited to energy storage, metabolic homeostasis, generation of heat, participation in immune functions and secretion of a number of biologically active factors known as adipokines. The most abundant of them is adiponectin. This adipocite-derived hormone exerts pleiotropic actions and exhibits insulin-sensitizing, antidiabetic, anti-obesogenic, anti-inflammatory, antiatherogenic, cardio- and neuroprotective properties. Contrariwise to its protective effects against various pathological events in different cell types, adiponectin may have links to several systemic diseases and malignances. Reduction in adiponectin levels has an implication in COVID-19-associated respiratory failure, which is attributed mainly to a phenomenon called ‘adiponectin paradox’. Ample evidence about multiple functions of adiponectin in the body was obtained from animal, mostly rodent studies. Our succinct review is entirely about multifaceted roles of adiponectin and mechanisms of its action in different physiological and pathological states.

## Abstract (ru)

Жировая ткань, состоящая из различных видов жира, является в организме одним из самых больших эндокринных органов, играющим множество ролей, которые включают, но не ограничиваются сохранением энергетических запасов, метаболическим гомеостазом, продукцией тепла, участием в иммунных функциях и секрецией целого ряда биологически активных факторов, называемых адипокинами. Самым обильно секретируемым из адипокинов является адипонектин. Этот вырабатываемый адипоцитами гормон оказывает плейотропное действие и обладает способностью повышать чувствительность к инсулину, а также демонстрирует антидиабетические свойства и эффекты противодействия развитию ожирения, воспаления, атеросклероза и, кроме того, еще и проявляет кардио- и нейропротективные свойства. С другой стороны, помимо вышеперечисленных защитных свойств и возможности предотвращения развития различных патологических процессов в разных типах клеток, адипонектин может быть связан с развитием ряда системных заболеваний и злокачественных опухолей. Снижение уровней адипонектина, как оказалось, наблюдается в том числе при дыхательной недостаточности, связанной с коронавирусной инфекцией COVID-19, что обусловлено в основном развитием феномена, называемого «адипонектиновый парадокс». Многочисленные доказательства многоликости функций адипонектина в организме были получены в ходе исследований на животных моделях, больше всего на грызунах. Наш краткий обзор полностью посвящен многофункциональной роли адипонектина и механизмам его действия при различных физиологических и патологических состояниях.

## BRIEF OVERVIEW OF ADIPOSE TISSUE STRUCTURE AND FUNCTIONS

An inexorable rise of obesity with over 650 million obese worldwide, driven mainly by changes in the global food system with an imbalance between energy intake and expenditure, was recognized by the World Health Organization (WHO) as a health problem of pandemic magnitude [1][2][3]. The topic has induced a tsunami of scientific interest in functions and roles of adipose tissues. In this connection, vigorous research in recent decades has provided ample evidence that adipose tissue widely distributed throughout the body is not only a dormant storage reservoir for fat cells but rather an endocrine multi-depot organ playing a crucial role in the control of energy homeostasis [4][5].

Depending on localization, adipose depots are broadly classified into subcutaneous, visceral (abdominal) and bone marrow fats [6]. Moreover, various depots have been shown to have distinct developmental origins from different compartments of mesoderm [7] and dissimilar depots also possess distinct functions due to composition of corresponding fat cell classes. According to their unique morphophysiological properties there are at least three different types of fat cells in the body, namely: white, brown and beige (also referred to as brite (from ‘brown-in-white)) [4][5][6][7][8].

White adipocytes composing the bulk of white adipose tissue (WAT) (recognized recently as an independent endocrine organ) can be subdivided into two subtypes: the ones that populate visceral depots in the abdomen called visceral WAT (vWAT) and their subcutaneous counterparts around the trunk, limbs and face called sWAT, respectively [8][9][10]. vWAT providing protective padding is distributed around internal organs and depending on its location is sub-classified into mesenteric, retroperitoneal, perigonadal and omental adipose tissue. sWAT is typically distributed on the hips, thighs, and buttocks but also inside the abdominal cavity underneath the skin as well as a scattered intramuscular fat [8]. WAT comprising as much as some 20% of body weight of normal adult humans is the main energy storing tissue in the form of packed in unilocular lipid droplets triglycerides [10]. The latter class of molecules stored anhydrously in its receptacle is highly energetic [4]. White adipocytes are flexible in their number and size, and are capable to expand well over 100 micrometers in diameter at times of overnutrition, while episodes of starvation with severe caloric restriction may result in a significant shrinkage of them [7][11][12].

In contrast to their white counterparts, brown adipocytes sharing a similar gene expression profile with myocytes [13] are rich in mitochondria and specialized in dissipating chemical energy in the form of heat through the combustion of various metabolites [14], defending mammals against hypothermia [4]. Brown adipose tissue (BAT) expressing constitutively high levels of thermogenic genes is distributed mostly in depots in the neck, scapulae, chest cavity and to some extent in the perirenal regions [15][16][17]. PET scanning in adult humans has allowed to identify presence of activated (by cold exposure) BAT at discrete anatomical sites including cervical, supraclavicular, pericardial, facial plane, between the subscapularis and pectoralis muscles, posterior to the brachial plexus and proceeding through thoracic and abdominal paraspinal sites, also with little perinephric activity [18]. Noteworthy, as few as 50 g of maximally stimulated brown adipose tissue could account for as much as 20% of total resting energy expenditure [19] and in this regards concepts of restoring energy balance in the body by means of activation of BAT in order to increase energy expenditure sound quite plausible [20].

Third type of adipocytes interspersed within the WAT and reputed as ‘mysterious’ [21] is beige, also known as “inducible brown” or “recruitable brown” [22–29]. The names imply that the cells exhibit overlapping but distinct properties of both white and brown adipocytes [6][7]. In their basal state (without stimulation by cold) this type of adipocytes is morphologically identical to its neighboring white counterparts [21], however upon reduction of temperature exposure or pharmacological activation of β-andrenergic receptors both de novo beige adipogenesis and the direct conversion (or transdifferentiation) of white into beige adipocytes occur [31]. Vice versa, the process of conversion of beige into white adipocytes takes place as a result of such stimuli as warming and high-fat diet [30][32][33]. Therefore, beige adipocytes can be induced from white-adipocyte-like phenotype to a brown-adipocyte-like one — the process called ‘browning of WAT’ [34].

All in all, activated brown and beige adipocytes releasing endocrine signals alter systemic energy metabolism by means of increasing substrate oxidation and energy expenditure [35]. Both brown and beige adipocytes can uptake glucose and fatty acids to produce heat, playing a pivotal role in regulating glucose and lipid metabolism in the whole body [36]. However, if one would compare pure clonal brown and beige cells, it appears that classical brown adipocytes have higher basal expression of uncoupling protein-1 (UCP-1) within the mitochondria and elevated uncoupled respiration, while beige cells have low basal UCP-1 expression (rather comparable to white adipocytes) and depressed uncoupled respiration [10]. Nevertheless, stimulation with a β-adrenergic agonist elevates UCP-1 to levels similar to brown fat cells, indicating that beige cells are uniquely bifunctional, namely: suited for energy storage in the absence of thermogenic stimuli, but capable of turning on heat production after receiving appropriate signals [37]. Moreover, experimental selective loss of brown fat causes compensatory induction of the beige one with concomitant restoring of body temperature and resistance to diet-induced obesity exhibiting significant overlap in functions [38].

Besides adipocytes per se, adipose tissues contain several other cell types and structures including but not limited to endothelial cells of vasculature, connective tissue matrix, nerve endings, as well as infiltrating immune cells [39]. Taken together all these components function as an integrated and well-orchestrated unit [40], responding to plethora of afferent signals from traditional hormone systems and central nervous system, and secreting itself multiple factors with paracrine, autocrine, juxtacrine and endocrine functions. Collectively these adipose-tissue-secreted hormones and cytokines were named “adipokines” and by now as many as more than 600 adipokines have been identified, excluding fatty acids and other metabolites [41]. Physiological functions of adipokines include regulation of glucose and lipid metabolism, body weight and appetite modulations, vascular homeostasis and blood pressure regulation, stimulation of angiogenesis and secretion of both classical cytokines and acute phase responders, etc. [42–44].

In the obese state, characterized by abnormal enlargement of WAT (hypertrophy and sometimes even hyperplasia of white adipocytes), systemic metabolic alterations, including hyperglycemia, insulin resistance and dyslipidemia occur, thus leading to the activation of proinflammatory pathways in adipocytes and enhancing the release of proinflammatory adipokines [39][44]. On the contrary to WAT, BAT activity is directed to protect against hyperglycemia, insulin resistance and dyslipidemia by means of releasing anti-inflammatory adipokines and metabolic substrates for oxidation [45][46]. However, sustained obesogenic insults inducing local proinflammatory signaling have detrimental effects interfering thermogenic function of BAT and beige recruitment and the browning of WAT, respectively [39]. Local action of proinflammatory cytokines and activation of inflammatory pathways attract infiltration of the associated immune cells, including proinflammatory macrophages, and all the aforementioned obesogenic machinery eventually contributes to systemic low-grade chronic inflammation [39][47] triggering several overlapping loops of vicious circles with lipotoxicity, insulin resistance, metabolic disturbances, endothelial dysfunction, etc. Moreover, imbalance in adipokines levels contributes to the development of autoimmune diseases [41][48] including diabetes mellitus type 1 [49], systemic lupus erythematosus [50][51], rheumatoid arthritis [52][53], ankylosing spondylitis [54], systemic sclerosis [55–57], and Behçet’s disease [58].

## ADIPONECTIN: STRUCTURE AND PLEIOTROPIC ACTIONS

The most abundant adipokine secreted by adipocytes is adiponectin first identified in both human and mouse forms and described in 1995 and 1996 by at least four independent groups [59–62]. Other names for this hormone used by the four research groups were gelatin-binding protein-28 (GBP-28), adipocyte complement-related protein (ACRP30), AdipoQ and apM1 [59–62]. We would like to focus our succinct review on this key collagen-like 244-amino-acid-long protein considered by many as a crucial ‘rescue hormone’ altering lipid and glucose metabolism and insulin sensitivity, stimulating mitochondrial biogenesis, exhibiting anti-inflammatory, anti-fibrotic, anti-thrombogenic, antioxidative, anti-atherogenic and cardioprotective properties, and also known to have links to several systemic diseases and malignances [63][64]. The polypeptide (a member of the complement 1q family) has four distinct domains including an amino-terminal region that target the hormone for secretion outside the cell, a variable one, a 65-amino-acid collagenous one and a carboxy-terminal globular domain [59][65][66]. The gene encoding adiponectin is located on chromosome 3q27, a region known as affecting genetic susceptibility to type 2 diabetes, metabolic syndrome, obesity and cardiovascular disease [67][68]. To date more than 50000 publications about this salutary adipocyte-derived circulating factor could be retrieved in PubMed, indicating that adiponectin has attracted much scientific attention because of its properties.

Adiponectin is synthesized intracellularly undergoing post-translational modifications by glycosylation and hydroxylation and then it is secreted from adipocytes into the bloodstream in three major isoforms, including low-molecular-weight (LMW) trimers (67kDa), middle-molecular-weight hexamers (MMW) (140kDa) and high-molecular-weight (HMW) oligomers (300kDa) [59][66][69–73]. However, a proteolytically cleaved form of globular adiponectin has also been found in the circulation [74][75]. Each of the multimers of adiponectin has been shown to exert distinct biological properties [68,76] with HMW as the predominant and the most active form of the protein playing a significant role in promoting glucose uptake and fatty acid oxidation [77], in facilitating insulin sensitivity [68][77–81] and in exerting its biological effects in the liver, muscles and endothelium [75][82]. Thus, normal oligomerization of adiponectin is critical to its physiologic action, while multimerization impaired by various factors is associated with insulin resistance, type 2 diabetes, obesity and arteriosclerosis [68][70][81–84].

Adiponectin exerts its beneficial pleiotropic effects through binding to three classes of its receptors, namely: AdipoR (with two subtypes: AdipoR1 and AdipoR2), T-cadherin and calreticulin. AdipoR1 expressed ubiquitously but most abundantly in skeletal muscle, while AdipoR2 predominantly expressed in the liver and both receptors contain a seven-transmembrane domain which are completely different from classic G-protein coupled receptors (GPCRs) and therefore they represent an entirely new class of receptor with presence of a zinc binding cite [85][86]. Basically, binding of adiponectin to AdipoR1 in skeletal muscle leads to the sequential activation of adenosine monophosphate-activated protein kinase (AMPK) pathway, then p38 mitogen-activated protein kinase (MAPK) pathway leading eventually to drastic increase in activation of peroxisome proliferator-activated receptor-α (PPAR-α) pathway and stimulating fatty acid oxidation and energy expenditure [87][88]. In the liver AdipoR1 also activates AMPK pathway suppressing hepatic gluconeogenesis and de novo lipogenesis and promoting fatty acid oxidation [85][88]. On the other hand, binding to AdipoR2 induces activation PPAR-α thus increasing expression of uncoupling protein-2 (UCP-2) which synergistically with AMPK signaling promotes fatty acid combustion [85]. Thereby, glucose and lipid metabolism, oxidative stress and inflammation are regulated [86]. Additionally, adiponectin through its receptors AdipoR1 and AdipoR2 induces ceramidase activation which leads to decreased levels of hepatic ceramide and promotes ceramide catabolism and therefore improves insulin sensitivity and slows down glucose production in the liver [89–91].

Mainly in the liver the full-length adiponectin ligand, as already mentioned above, functions as an insulin sensitizer signaling through both AMPK-dependent (by controlling hepatic ketogenesis, cholesterol synthesis and triglyceride synthesis [92]) and AMPK–independent pathways to suppress glucose production. At the same time, proteolytically processed globular form of adiponectin signals through AMPK pathway activation, promotes fatty acid oxidation chiefly in skeletal muscle facilitating glucose uptake. Additionally, the trimer and hexamer forms of adiponectin can act in the central nervous system (CNS) regulating appetite, energy expenditure and even producing antidepressant effects [68].

Yet another key molecules playing a crucial role in various signaling pathways of cell proliferation, chromatin remodeling, endosomal trafficking, cell survival, metabolism and apoptosis are adaptor proteins containing the pleckstrin homology domain, phosphotyrosine binding domain and leucine zipper motif (APPLs) [93]. APPLs isoforms– APPL1 and APPL2 have been found to interact directly with adiponectin receptors AdipoR1 and AdipoR2 and to transduce adiponectin signaling to enhance glucose uptake and lipid oxidation therefore mediating the crosstalk between insulin and adiponectin pathways [93].

A unique member of glycosylphosphatidylinositol-anchored cadherin superfamily lacking the transmembrane cytoplasmic and domains T-cadherin is known to be highly expressed in endothelial cells; in smooth muscle, skeletal muscle and cardiac muscle cells; in neurons and in mesenchymal stem/stromal cells (MCSs) [94]. Moreover, it was found that T-cadherin is one of the most abundantly expressed proteins on the cell surface of MCSs [95]. Noteworthy, T-cadherin has been identified as a receptor for hexameric and HMW forms of adiponectin but not for trimeric and globular ones [96]. Binging of adiponectin to T-cadherin leads to endocytosis into microvesicular bodies (MVBs) and then their release as exosomal cargo which results in increased efflux of ceramide in exosomes with concomitant decrease of cellular ceramide levels [97]. All in all, ceramide lowering effects of adiponectin/T-cadherin is an important mechanism in protection against various organs and tissues damage, and against metabolic disturbances, while T-cadherin is a key binding partner for adiponectin [98]. In the heart T-cadherin was found to be crucial in mediating adiponectin effects against ischemia-reperfusion injury [99].

Calreticulin, a multifunctioning soluble protein binding Ca2+ ions and expressed on both the phagocytic and the apoptotic cell surface, plays a pivotal role in apoptotic debris disposal through its interactions with CD95 [100][101]. Binding of adiponectin to calreticulin on the macrophage cell surface facilitates the uptake of early apoptotic cells by macrophages [102]. Therefore, adiponectin accounting for as much as 0.01% of total plasma protein [103] protects the organism from systemic inflammation by promoting the phagocytosis of early apoptotic cells by macrophages through a receptor-dependent pathway involving calreticulin but not through AdipR1, AdipoR2 and T-cadherin [102]. Hence, the latter three receptors are involved in mediation of the metabolic properties of adiponectin, while calreticulin mainly controls aspects of adiponectin’s anti-inflammatory actions.

Human adiponectin despite it abundant presence in serum (nearly three orders of magnitude higher than other adipokines) has a half-life of approximately 75 minutes [104] and its plasma levels are higher in women compared to men [105] which attributed to differential body fat and fat types distribution between males and females [106], as well as to selective inhibition by testosterone of the secretion of HMW adiponectin [107]. Accordingly, it has been shown that females have higher proportion and absolute amount of HMW and hexamers, and a significantly lower the proportion of trimers [104].

It is also worth mentioning, that approximately 42% of the total adiponectin pool in the human body resides in the extravascular compartment but rapid postprandial redistribution between extra- and intravascular compartments occurs [104].

## ADIPONECTIN AND VARIOUS DISEASES

Ample epidemiological evidence indicates that individuals with obesity (particularly with morbid obesity), diabetes, metabolic syndrome, hypertension and coronary heart disease have decreased levels of adiponectin, with especially low HMW adiponectin [68][108–112]. Moreover, decreased ratio of HMW to total adiponectin strikingly correlates with angiographic coronary atherosclerosis severity [113], while adiponectin protects against all stages of atherosclerotic plaque formation [114]. Obesity and cardiovascular disease (CVD) are recognized as chronic low-grade inflammatory conditions [115]. This low-grade inflammation termed “metabolic inflammation” or metainflammation is responsible for the decrease of insulin sensitivity through activation of JNK, NF-κB and inflammasome pathways; through concomitant production of proinflammatory cytokines and proinflammatory macrophages infiltration [116–118]. In CVD chronic inflammation of the vessel wall results from the transendothelial passage of cholesterol-rich atherogenic Apo-B lipoproteins from plasma into the intima, local secretion of a copious amount of reactive oxygen species (ROS) and production of oxidized lipoproteins leading ultimately to endothelial cell apoptosis, which induces infiltration by macrophages, mast cells and T-cells [115][119]. As we already mentioned above, such sort of vicious circle between oxidative stress and inflammation takes place not only in the arterial wall, but also in adipose tissues impairing adipocytes maturation, insulin action and adipokines signaling [119]. Considering the fact that adiponectin is negatively correlated with adiposity and that it has anti-inflammatory properties as well as inverse relationship with several inflammatory markers, this adipocytokine was proposed to be a link between obesity and inflammation [120][121]. Moreover, it was epically proclaimed as a ‘guardian angel’ against the pathophysiology of obesity and diabetes [120]. In general, its protective effects against insulin resistance and inflammation are due to this adipokine’s capacity to ameliorate lipid and simple carbohydrates profiles [122] and to its ability to inhibit monocytes adhesion, transformation of macrophages into foam cells and lipid accumulation in macrophages in the vessel wall via reduction of the expression of adhesion molecules and scavenger receptors [123][124]. It also inhibits proliferation of vascular smooth muscle cells (VSMCs) maintaining their contractile phenotype [125]. Adiponectin exerts its protective effects on cardio-vascular system partly through the increase of nitric oxide (NO) production, endothelium-dependent vasodilation [126] and concurrent stimulation of neovascularization [127]. It has also been demonstrated that in endothelial cells adiponectin decreases TNF-α-induced expression of intercellular adhesion molecule-1 (ICAM-1) and NF-κB activation [128][129]. Yet another pleiotropic effect of this adipokine is limiting of monocytic microparticle-induced endothelial activation at least in part through the AMPK, Akt and NF-κB signaling pathways [130]. It also inhibits collagen-induced platelet aggregation and abrogates C-reactive protein mRNA and protein synthesis and secretion via upregulation of AMPK and downregulation of NF-κB pathways [131]. It is therefore evident that adiponectin protects cardiovascular tissues under conditions of stress through several mechanisms, including inhibition of proinflammatory and hypertrophic responses as well as through stimulation of endothelial cell responses [132]. Thus, it attenuates excessive inflammatory responses to multiple stimuli regulating several signaling pathways in a variety of cell types and tissues [121].

Accordingly, adiponectin has been shown to be protective against fatty liver disease increasing hepatic insulin sensitivity and attenuating liver inflammation and fibrosis [133], therefore exerting antisteatotic, anti-inflammatory and antifibrogenic effects [134], as well as it was reported to be anti-apoptotic on pancreatic β-cells [135]. In the kidney adiponectin via AMPK pathway activation inhibits NADPH oxidase which results in preventing of podocytes injury, improving their dysfunction, inhibiting inflammation, fibrosis and oxidative stress [136][137]. However, high serum adiponectin levels were found in chronic kidney disease due to impaired urinary excretion, which can be explained by its clearance via glomerular filtration [138][139]. Moreover, after successful kidney transplantation in patients with chronic renal failure significant reduction of adiponectin serum concentration was found, implying that the kidney plays an important role in biodegradation and/or elimination from the circulation of adiponectin [139][140] and making this protein a biomarker of renal disease outcomes [139].

As for cellulite, a complex multifactorial cosmetic disorder of the subcutaneous fat layer and the overlying superficial skin on the thighs and buttocks, it has been demonstrated that adiponectin expression is significantly reduced in the subcutaneous areas affected by cellulite [141]. Since adiponectin is known as a humoral vasodilator, it might contribute to the altered microcirculation in cellulite areas.

Hypertension is just one more pathologic condition where adiponectin was also found to play a role modulating blood pressure. In patients with essential hypertension plasma adiponectin levels were significantly lower than in normotensive subjects [142]. As we already described above, adiponectin protects against endothelial dysfunction via several regulatory pathways. One pathway in endothelial cells involves enhancing of nitric oxide synthase (eNOS) activity and NO production via AdipoR1/R2-AMPK-endothelial signaling and the other one is via cyclooxygenase-2 (COX-2) expression and prostaglandin I2 (PGI2) production through calreticulin/CD91-dependent Akt signaling [143]. Furthermore, adiponectin is able to attenuate the phenotype of macrophages M1 (M1 activity is known to inhibit cell proliferation and tissue damage) and to promote the phenotype of macrophages M2 (M2 activity is known to promote cell proliferation and tissue repair). Therefore, it is plausible that adiponectin exerts protective actions on vascular functions at least in part through improving functions (excuse the pleonasm here) of macrophages and endothelial cells [143].

Adiponectin is also among hormones controlling the interaction between energy balance, fertility and reproduction in humans. In males it modulates several functions of both somatic and germ cells including steroidogenesis, proliferation, apoptosis and oxidative stress, while in females adiponectin is involved in the control of steroidogenesis in ovarian granulosa and theca cells, oocyte maturation, and embryo development [144]. At the testicular level autocrine/paracrine actions of adiponectin have been demonstrated to promote spermatogenesis and sperm maturation [145]. Additionally, adiponectin receptors were found in placental and endometrial cells, suggesting that it might play a crucial role in embryo implantation, trophoblast invasion and fetal growth [144]. Therefore, adiponectin has obviously beneficial effects on both female and male reproductive functions.

Contrastingly to the well-known canonical anti-inflammatory effects of adiponectin, there is a vast literature describing its non-canonical proinflammatory properties in several diseases that are unrelated to increased adipose tissue [146]. These are inflammatory/autoimmune disorders including rheumatoid arthritis (RA), systemic lupus erythematosus (SLE), chronic kidney disease (CKD), inflammatory bowel disease (IBD), type 1 diabetes (T1D) and chronic obstructive pulmonary disease (COPD) [147]. Adiponectin, a member of C1q/TNF-related protein superfamily (CTRP), selectively binds several anionic phospholipids and sphingolipids, including phosphatidylserine, ceramide-1-phosphate, glycosylceramide and sulfatide via the C1q domain in liposomes, low-density lipoproteins, cell membranes and plasma, suggesting that it can function facilitating opsonization of lipids [148]. Furthermore, via promotion of proinflammatory responses by CD4+ T-cells and macrophages it can also stimulate production of several cytokines and chemokines such as INF-γ and TNF-α secretion, respectively [149].

It has been shown that in fibroblast-like synoviocytes (FLS) of RA patients adiponectin stimulates production of proinflammatory factors including IL-6, IL-8, PGE2 [150] and enhances the production of vascular endothelial growth factor (VEGF) and matrix metallopeptidases (MMPs), while in osteoblasts it leads to increase in levels of IL-8, MMP-9 and tartrate-resistant acid phosphatase (TRAP) and in RA-induced human bone tissue it inhibits osterix expression, elevates osteoprotegrin mRNA expression, decreasing mineralization capacity of osteoblasts and increasing resorptive activity of osteoclasts, which eventually may lead to joint destruction and hinders bone formation [151][152].

In SLE, an autoimmune disease characterized by the immune system attack on its own tissues and organs, presence of antibodies to nuclear and cytoplasmic antigens, multisystem widespread inflammation and protean clinical manifestations, a positive association of serum adiponectin levels with SLE-related atherosclerotic plaques formation and a strong correlation of urine adiponectin concentrations with lupus nephritis were found [153].

It is well established that adiponectin is present in the kidneys mainly in the arterial and capillary endothelium as well as in the smooth muscle cells. Patients with CKD, especially with end stage renal disease (ESRD) have significantly elevated adiponectin levels chiefly with elevation of HMW form of adiponectin and its concentration was found to inversely associate with estimated glomerular filtration rate (eGFR) [154]. Considering the fact that the kidneys play an important role in the biodegradation and clearance of adiponectin via glomerular filtration, its higher serum levels may be due to impaired urinary excretion [139][155]. Furthermore, there is a disruption in the normal adiponectin signaling through AMPK and several other pathways as a result of noxious effects of the uremic milieu.

As for IBD, a chronic idiopathic inflammatory condition comprised of two major disorders: ulcerative colitis and Crohn disease, adiponectin signaling through AdipoR1 has been shown to exacerbate colonic inflammation through two possible mechanisms, namely: 1). increased production of proinflammatory factors like IL-6, MIP-2 and COX-2 (the latter promotes the production of PGE2 from arachidonic acid in the colon); and 2). enhanced neutrophil chemokine expression and recruited neutrophils into the colonic tissue which in turn increase inflammation [156].

In T1D patients despite elevated mean both total and HMW adiponectin levels decreased insulin sensitivity compared with nondiabetic controls was observed, suggesting an increased set point or dysregulation of adiponectin function in the subjects with T1D [157]. These findings are consistent with a relative adiponectin resistance among this cohort of patients and support the proposed hypothesis that factors unrelated to adiponectin contribute to decreased insulin sensitivity in this population.

Adiponectin and all of its known receptors including AdipoR1, AdipoR2, T-cadherin and calreticulin are expressed on multiple cell types in the lungs, moreover adiponectin has also been isolated from BAL fluid [158]. Serum adiponectin concentrations were found to be significantly higher in patients with COPD particularly in the phase of exacerbation, hence the hormone might represent an indirect marker of low-grade systemic inflammatory response and a severity of COPD [159]. Elevated levels of serum adiponectin have also been revealed in patients with cystic fibrosis (CF) with normal nutrition which may be attributed to the energy deficit inherent to the disease. On the contrary, decreased adiponectin levels were observed among malnourished patients with CF which probably might be explained by lipodystrophy-like body fat reduction [160]. Furthermore, sputum adiponectin levels were higher in CF patients with pancreatic insufficiency versus cases of CF with pancreatic sufficiency [161]. In general, these data indicate that under some chronic inflammatory conditions lasting for prolonged periods, adiponectin may exacerbate inflammation in several cell types and tissues.

Noteworthy, dissimilar to the above mentioned autoimmune conditions, adiponectin can act on keratinocytes and naïve T-cells and therefore has its rather an anti-inflammatory role in the pathogenesis of one more autoimmune disorder psoriasis by increasing the production of IL-10, while inhibiting the production and activity of IL-2, Il-6, IL-8, IL-17, IL-22, TNF-α and IFN-γ [162]. Moreover, adiponectin similarly to adipocytes is expressed in human sebaceous glands affecting the homeostasis of the dermis. Patients with psoriasis have lower plasma adiponectin levels which thereby may lead to increased production of proinflammatory cytokines and lack of anti-inflammatory ones which eventually worsen the severity of their skin lesions. In keratinocytes the decrease in adiponectin levels has been shown to result in the reduced E2F1 gene activation through AMPK pathway promoting the proliferation of keratinocytes and inducing abnormal cell apoptosis thus interacting with the infiltration of the inflammatory response and leading to psoriasis [162].

Taking into account antiangiogenic and tumor-growth limiting properties of adiponectin [163], it would be a significant gap in our review if we skip the topic of its roles in cancer. Noteworthy, adiponectin receptors are expressed in a plethora of malignant tissues, while activation of the receptors limits the proliferation of cancer cells in vitro [164]. Several studies have demonstrated an inverse association between circulating adiponectin levels in vivo and the risk of malignances associated with obesity and insulin resistance videlicet, endometrial cancer [165], postmenopausal breast cancer [166], leukemia [167] and colon cancer [168]. Moreover, low adiponectin levels have been associated with prostate and gastric types of cancer [169][170]. Reduced levels of plasma adiponectin can potentially contribute to carcinogenesis through altered effects of TNF-α and VEGF leading to promotion of cellular proliferation and inhibition of apoptosis in various cell types [163]. Furthermore, adiponectin may selectively bind several mitogenic growth factors at a pre-receptor level sequestrating them and thus exerting an antiproliferative effect [171]. Lack of adiponectin can therefore contribute to the development of tumors, while stimulation of AMPK pathway by adiponectin [172] may instead inhibit growth and/or survival of cancer cells [173]. Anticancerogenic effects of adiponectin may also involve AMPK-independent multiple pathways including blocking of MAPK pathway activation partly through the increase of NO production and also through attenuation of actions of oxidized low-density lipoprotein (OxLDL), which reduce cell proliferation [172][174]. Another possible pathway involvement is activation of caspases cascade by adiponectin eventually leading to cell death [175]. More intracellular signaling pathways involved in adiponectin-mediated inhibition of cancerogenesis include: NF-κB, PI3K/Akt/mTOR, activation of PKA, inhibition of β-catenin and in particular sphingolipid metabolic pathway [164]. Lastly, it was reported that adiponectin stimulates c-Jun N-terminal kinase (JNK) activation and also drastically suppresses STAT3 pathway in prostate and hepatocellular carcinomas known to express AdipoR1 and AdipoR2 receptors, and thus may affect the pathogenesis of these types of cancer [176]. As for the already described above nonclassical potential adiponectin receptor T-cadherin, it has been reported to be expressed on tumor-associated endothelial cells [177] therefore affecting tumor angiogenesis directly. Some authors hypothesized that since T-cadherin lacks an intracellular domain responsible for signal transduction, it may act as a coreceptor by competing with AdipoR1 and AdipoR2 receptors for binding of adiponectin hence restricting AdipoR1/R2 signaling and may therefore induce interference with signal transduction events [178]. It is also worth mentioning that influence of adiponectin in endocrine cancer cells may depend to some extent on paracrine interactions between tumor cells and neighboring adipocytes as these types of cells are often in close proximity to each other and one of such examples is the case of breast cancer, when adiponectin negatively modulates aromatase activity in adipocytes affecting estrogen production and reducing estrogen receptor alpha (ERα) stimulation in adjacent cancer cells [179]. However, adiponectin effects on breast cancer cell growth may diverge depending on ERα expression, when in ERα-negative cells it exerts anti-proliferative properties while in ERα-positive ones it promotes cancer cell proliferation [180]. Significantly decreased serum concentrations of adiponectin have been also demonstrated in most forms of thyroid carcinoma with papillary one in particular presumably due to indirect effects of adiponectin through regulation of insulin sensitivity and metabolism [181]. Nevertheless, this correlation with low serum levels of the adipokine was not found in medullary thyroid cancer [182]. In endocrine malignances it was reported that adiponectin may be able to suppress several important processes leading to metastatization, including adhesion, invasion and migration of, for instance, breast cancer cells [183]. The reduction of cancer cell migration and invasion by adiponectin at least in part occurs through the AMPK/Akt pathway [184]. All in all, adiponectin appears to play numerous roles in initiation, promotion, progression and prevention of various types of cancer exerting its antitumorigenic effects directly on cancer cells by stimulating receptor-mediated signaling pathways and also indirectly by modulating insulin sensitivity at the target tissue site, by influencing tumor angiogenesis and by regulating inflammatory responses.

## ADIPONECTIN AND SARS-COV-2

At last, while reviewing multifaceted roles of adiponectin, it is virtually impossible not to mention the most pernicious problem of the last couple of years, namely the ongoing global pandemic of coronavirus disease 2019 (COVID-19), caused by the highly pathogenic virus SARS-CoV-2, and a potential implication of adiponectin. As an efficient antiviral response is driven by T-helper lymphocytes (LTh) with a specific polarization such as LTh1 and LTh2, it is now apparent that adiponectin may contribute to promoting primarily LTh1 polarization, which in turn triggers anti-viral inflammation [185]. Nevertheless, in the publication of van Zelst C.M. et al. [186] in a relatively small number of patients not requiring intubation no difference in levels of serum adiponectin between COVID-19 negative and COVID-19 positive patients was found. On the contrary, in another study investigating a role of adipose tissue in COVID-19 onset it has been demonstrated that adiponectin levels were higher in patients with severe cases of the infection as compared to mild and moderate ones [187]. Taking into consideration a correlation of adiponectin and IL-6 serum concentrations, authors suggest an attempt of adipose tissue to counteract inflammation from the beginning of the infection [187]. Moreover, in the publication with eloquent title “When two pandemics meet: why is obesity associated with increased COVID mortality?” special emphasis is given on the role of significantly decreased circulation levels of adiponectin in obese patients which facilitates an exaggerated inflammatory response in the capillaries of the lungs and negatively affects survival in this cohort of ‘double pandemic’ patients [188]. Accordingly, markedly reduced levels of adiponectin have been reported in patients with COVID-19 respiratory failure even after adjustment for BMI which implies that COVID-19 infection may independently itself reduce adiponectin levels in humans with respiratory failure [189]. The authors speculate that this fact has a specific implication for patients with obesity, which is a major risk factor for a negative clinical prognosis, and hypothesize that individuals with persistently low adiponectin levels are more prone to develop COVID-19-associated respiratory failure [189]. Interestingly, adipose tissue itself can serve a viral reservoir as the expression of angiotensin-converting enzyme 2 (ACE2) — the functional receptor for severe acute respiratory syndrome coronavirus 2 (SARS-CoV-2), is known to facilitate viral penetration into target cells [190]. Further upon tissue penetration of the virus, viral RNAs are released, triggering so called ‘cytokine storm’ characterized by excessive release of IL-1, IL-6, IL-18, IFN-γ, TNF-α and leading to overwhelming systemic inflammation, hyperferritinemia, hemodynamic instability, multi-organ failure, and if left untreated, ultimately to death [191–193]. Meanwhile, ACE2 was observed to be upregulated in adipocytes of patients with obesity and diabetes and this dysfunctional adipose tissue may undergo increased fibrosis (and adiponectin is potentially antifibrotic and it is likely able to restore normally functioning adipose tissue) [190]. Hence, both diseases are potential comorbidities for COVID-19 significantly aggravating its clinical course [190]. Moreover, taking into account the fact that the virus may reduce functional circulating ACE2 and one of the tissues where ACE2 is abundantly expressed is pancreatic β-cells, SARS-CoV-2 might target and disturb normal pancreatic function, alter glucose metabolism and induce de novo type 2 diabetes (T2DM ) [194]. Accordingly, upon SARS-CoV-2 infection pancreatic beta-cells have been demonstrated a lower expression of insulin and higher expression of alpha and acinal cell markers, including glucagon and trypsin suggesting cellular transdifferentiation [195]. Therefore, and even more striking, pancreatic injury as a result of COVID-19-diabetes has reciprocally bidirectional relationship [196], when in one direction T2D may increase risk of the infection and in the other one active T2D is an independent predictor of morbidity and mortality in patients with coronavirus infection [197]. However, an alternative opinion questioning the idea of direct infection and injury of β cells by SARS-CoV-2 also exists in accordance with which β-cell damage is attributed to a “bystander” effect in which the infection leads to damage of surrounding tissues that are essential for β-cell survival and function, namely the pancreatic microvasculature and exocrine tissue [198]. Contrastingly to this opinion direct SARS-CoV-2 viral infiltration of the islets has been reported using immunofluorescence and electron microscopy techniques, although ACE2 expression was found to be more common in endothelial vasculature than in β-cell while adiponectin (as we mentioned above adiponectin is known to be anti-apoptotic on pancreatic β-cells [135]) levels were also reduced [199]. All in all, it is now apparent that reduction in adiponectin levels may negatively affect its ability to reduce the amount of proinflammatory cytokines and ability to stimulate the content of anti-inflammatory ones (for instance, IL-10), and may also induce impaired expression of AipoR1, AdipoR2 and, above all, of T-cadherin [199]. The latter is a crucial molecule as any disturbances in its expression or functioning of T-cadherin or the binding of adiponectin correlate with endothelial dysfunction and pulmonary and cardiovascular pathologies [200]. As we already described above in details in such circumstances it would be a decreased efflux of ceramide in exosomes with concomitant increase of cellular ceramide levels impairing the mechanism of protection against various organs and tissues damage, and against metabolic disturbances. Noteworthy, in newborns where a so called ‘neonatal hyperadiponectinemia’ is observed at delivery, it may positively influence on the prognosis for COVID-19 infection [201].

## FACETS OF ADIPONECTIN PARADOX

Paradoxically, accumulating reports of results of several meta-analyses demonstrated that elevated serum levels of both total and HMW adiponectin have been positively associated with both cardiovascular and, what is quite surprising and confusing, even with all-cause mortality rate in the population above 65 years of age [reviewed in 202]. This controversial and ambiguous relationship has been called ‘adiponectin paradox’. One of the explanations for such phenomenon is based on the direct correlation between natriuretic peptides (NPs), well-known as predictors of all-cause mortality [203] and adiponectin levels. NPs promote adiponectin secretion and therefore increase its serum concentrations [204]. In such circumstances adiponectin simply serves as a marker of elevated NPs. Other plausible explanation at least in patients with cardiovascular risk and particularly with concomitant metabolic disorders is impaired liver function affecting adiponectin’s degradation in the liver [205,206]. Hence, it is rather a secondary feature of metabolic disorders than a primary cause of CVD. Besides, despite the fact that mostly HMW form of adiponectin exerts its cardioprotective properties, a majority of studies mechanistically evaluated total adiponectin instead of its quality [207]. Nevertheless, in those studies where HMW form was measured, its comparable elevation and correlation with incidence of mortality in patients with chronic heart failure have been found [208][209]. There was also reported that elevated adiponectin levels predicting cardiovascular mortality could be observed in men but not women [210]. The latter may be attributed to gender differences in adipose tissue distribution with so called «pear shape» with predominance of subcutaneous WAT in women and «apple shape» body habitus with more visceral WAT in men [211]. One more hypothesis that may at least in part explain the phenomenon is a possibility that increase of adiponectin is a failing attempt of the body to protect individuals with high risk of mortality [210], as pathological adiponectin resistance with its receptors downregulation develops in metabolically active organs including the adipose tissue, the heart, the liver, the vasculature, etc. [212]. Accordingly, augmented adiponectin production, for instance, in patients with advanced heart failure is assumably a compensatory mechanism due to the development of adiponectin resistance alike insulin resistance and hyperglycemia, which drive hyperinsulinemia [212]. It is now apparent that adiponectin resistance most commonly develops in obesity, diabetes and heart failure, when it is accompanied by decreased receptors expression, reduced receptor sensitivity and impaired downstream signaling [212]. Besides, high serum adiponectin levels were noted in patients with a reduced kidney function and the latter is an important cause of premature death [213,214]. We already described above in details the fact that significant elevation in adiponectin levels has been observed in patients with ESRD (chiefly with elevation of HMW form) and its concentration inversely associates with eGFR [154]. Therefore, higher serum levels of adiponectin may be due to impaired urinary excretion [155] and, perhaps, microvascular damage seen in nephropathy and other pathologic states [215]. However, serum adiponectin is obviously just a biomarker of renal dysfunction rather than a true risk factor of CKD progression [155]. Nevertheless, in maintenance hemodialysis (MHD) patients with few comorbidities high blood adiponectin levels were rather good prognostic markers, while in MHD population with a high comorbidity burden elevated levels of the hormone were associated with poor clinical outcomes and increased mortality risk [216]. Body mass index (BMI) was also suggested to be a confounder on the association between adiponectin and mortality, although studies addressing the issue have demonstrated discordant results [reviewed in 202]. Notably, ‘adiponectin paradox’ may play a detrimental role not only in circulatory disorders like CKD and chronic heart failure (CHF) but the paradox might be applicable to neurodegenerative diseases. Accordingly, it has also been proposed that adiponectin plays an important role in the pathogenesis of Alzheimer’s disease (AD) [217][218]. It was quite unanticipated since adiponectin has been shown previously to ameliorate neuropathological features in mouse models of neurodegeneration [217]. Anyway, it was reported that adiponectin is associated with the severity of amyloid accumulation and cognitive decline, while AD is associated with hyperadiponectinemia [217–219]. Elevation of adiponectin might be a consequence of compensatory feedback to the decreased activity of insulin/IGF-1 receptor signaling pathway in the neurodegenerative conditions [217]. During the progression of the disease adiponectin may increase and sequester by tau- the microtubule-associated protein forming insoluble filaments that accumulate as neurofibrillary tangles in AD, eventually leading to neurotoxic protein aggregation in the brain, synaptic loss and neuronal cell death [218]. Thus, ‘adiponectin paradox’ mainly observed in aging-associated chronic diseases with concomitant metabolic dysfunction, including neurodegenerative and circulating diseases may be considered as playing an important role phenomenon in age-associated conditions and also in the emerging field of geroscience [218]. Additionally, higher serum adiponectin concentrations are associated with development of cancer and cancer-related deaths in T2DM patients- one more implication of ‘adiponectin paradox’ and this was reported just recently [220].

Moreover, a link between ‘adiponectin paradox’ and SARS-CoV-2 infection and its chronic complications has also been demonstrated lately [221]. Curiously, antagonistic pleiotropy was found. Accordingly, SARS-CoV-2 may augment both chronic inflammation and adiponectin serum levels and this plays a beneficial role contributing to more efficient combating acute infection in younger ages representing a kind of hormesis. Conversely, in aging population SARS-CoV-2 induction of ‘adiponectin paradox’ is detrimental [221].

On the whole, the paradox is at the forefront of the research, but publications concerning the matter are sometimes contradictory. Noteworthy, the authors of the commentary «Adiponectin: good, bad or just plain ugly?» aptly noticed that conflicting data in the literature (quote) «may reflect our inability to paint a complete picture» [215]. Hopefully, the enigmatic phenomenon of adiponectin paradox can be solved in the nearest future.

Noteworthy, when our manuscript was close to its final layout, a new plausible explanation for 'adiponectin parodox' was proposed by Hans O. Kalkman in his review article in December 2021 issue of Pharmaceuticals [233]. Accordingly, T-cadherin, also know as a co-receptor for AdipoRs, is a key culprit for the paradox. T-cadherin, which is anchored to the cell membrane through a glycosylphosphatidylinositol moiety, can be specifically cleaved by phospholipase D (GPI-PLD). In several pathological conditions when excessive elevation of circulating GPI-PLD levels is observed, this leads to an increased hydrolysis of membrane-bound T-cadherin and, therefore to a reduced adiponectin sequestration by responsive tissues as wells to impaired adiponectin signaling and consequently to augmented adiponectin levels in circulation. Moreover, since HMW-adiponectin/T-cadherin system is responsible for ceramide removal from the cell [97], any dysfunction in the system can further aggravate impaired signaling, including the insulin resistance. In such circumstances metformin, acting on adiponectin pathway and inducing AMPK activation downstream of adiponectin receptors, can be a therapy of choice [233].

## RODENT STUDIES OF ADIPONECTIN

Incredible amount of data about adiponectin’s multiple roles in the body was obtained from pioneering studies carried out in rodent models. Excessively detailed description of such sort of research is not the main purpose of our review, anyway we would like to portray the topic with a few strokes. For instance, in studies of transgenic globular adiponectin ob/ob mice partial amelioration of insulin resistance and diabetes, but not of obesity were found implying that sustainably high levels of globular adiponectin have insulin-sensitizing effect, which is independent of WAT [222][223]. Moreover, in constitutively high cholesterol levels apolipoportein E (apoE)-deficient/globular adiponectin transgenic mice globular adiponectin could inhibit development of severe atherosclerosis in the aorta as compared to apoE-deficient control transgenic animals [223]. This results were corroborated later in apoE-deficient knockout mice [224]. In adiponectin knockout mice insulin resistance, glucose intolerance, hyperlipidemia, hypertension and other metabolic syndrome-related traits were found as well as more abundant neointimal formation in response to injury unveiling the protective role of adiponectin [225][226]. In transgenic mice with the collagenous domain of adiponectin deletion elevation of circulating adiponectin levels leading to improved insulin sensitivity was reported [227].

Besides, it has been proved that sustained peripheral expression using recombinant adeno-associated virus (rAAV) vectors of transgene adiponectin offsets the development of diet-induced obesity [228]. In the latter study conducted at the University of Florida, where one of the authors of the current review took part as an integral part of the collaborative research team, the long-term (up to 280 days) expression of AAV-Acrp30 vectors encoding mouse Acrp30 cDNA was tested after intramuscular and intraportal injections in female Sprague-Dawley rats. It is worth mentioning that rAAV is a prototypical gene therapy vector with excellent safety profiles, broad host range and ability to transduce differentiated cells [229]. So, using rAAV for Acrp-30 gene delivery, it has been demonstrated that a single intraportal injection of 1012 physical particles of the vector resulted in sustained reduction of body weight, concomitant with the reduction of daily food intake as compared to the control group with mock rAAV in which diet-induced obesity (DIO) has developed. A possible and certainly very intriguing explanation for such offsetting of DIO is based on modulations of hepatic gluconeogenesis and lipogenesis as a result of the reduction of the expression of the two key genes in the liver, namely: phosphoenolpyruvate carboxykinase (PEPCK) and sterol regulatory element-binding protein 1c (SREBP-1c) with signaling of the both through AMPK pathway [228]. Nevertheless, expression of Acrp30 in muscles resulted in significantly weaker effect incomparable to the one with the expression of the gene in the liver. Moreover, the most unexpected finding of the study was relatively low plasma concentration of murine Acrp30, which was two orders of magnitude lower than physiological levels of endogenous rat Acrp30. Obviously, the main effect was achieved at the hepatic level due autocrine and/or paracrine interactions of exogenous Acrp30 with AdipoR2 [228].

Furthermore, Li X. et al. in their study have demonstrated that both globular and full-length adiponectin reverses high-fat diet-induced insulin resistance in mice through decreased PKCε activation in the liver and decreased PKCε/PKCθ activity in muscles [230].

In another study in rats it was found that globular adiponectin resistance develops independently of impaired insulin-stimulated glucose transport in muscles of high-fat diet rats [231]. One more striking result was achieved after restoration of normal adiponectin concentrations in obese pregnant mice, which has led to prevention of placental dysfunction, fetal overgrowth and metabolic syndrome development as well as prevention of cardiac dysfunction in the adult offspring [232].

## CONCLUSION

Adiponectin epically proclaimed as a ‘guardian angel’ against the development of obesity and diabetes is a crucial ‘rescue hormone’ secreted mainly by white adipose tissue (and by placenta in pregnancy) and regulating lipid and glucose homeostasis, insulin sensitivity, energy balance and stimulating mitochondrial biogenesis. It is now apparent that adiponectin is the most abundant protein synthesized and secreted by mature adipocytes, however its serum levels inversely correlate with increases in adiposity, especially with its central subtype. The preponderance of evidence suggests that adiponectin is a pivotal link between adiposity, insulin resistance, inflammation and atherosclerosis. The hormone, protecting against various pathological events in different cell types, exerts pleiotropic actions and exhibits anti-inflammatory, antifibrotic, antithrombogenic, antioxidative, antinitrative, antiatherogenic, cardio- and neuroprotective properties on the one hand, may also have links to several systemic diseases and malignances on the other. Furthermore, it may even have an implication to severity of SARS-CoV-2 depending on the age and the development of the phenomenon called ‘adiponectin paradox’. Adiponectin exerts its both beneficial and detrimental effects via normal or impaired signaling through its receptors: AdipoR1 and AdipoR2, T-cadherin and calreticulin. AdipoR1 is expressed abundantly in skeletal muscle and endothelial cells; AdipoR2 — predominantly in the liver; T-cadherin is highly expressed in endothelial cells, in smooth muscle, skeletal muscle and cardiac muscle cells, in neurons and in mesenchymal stem/stromal cells; while calreticulin, endoplasmic reticulum luminal Ca2+-buffering chaperone, found in virtually every cell of the body (except erythrocytes), is responsible for endothelial anti-inflammatory and vasculoprotective effects of adiponectin. It is worth mentioning, that ample evidence about multiple favorable physiological as well as pathological roles of adiponectin has been obtained from studies carried out in animal, mainly rodent, models. Accumulating reports demonstrate that modulation of levels of adiponectin and its isoforms, normalization of its signaling patterns may lead to novel therapeutic approaches for many diseases. However, significant knowledge gaps remain and it is apparent, therefore, that further in-depth research in this field is imperative.

## ДОПОЛНИТЕЛЬНАЯ ИНФОРМАЦИЯ

Источники финансирования. Работа выполнена по инициативе авторов без привлечения финансирования.

Конфликт интересов. Авторы декларируют отсутствие явных и потенциальных конфликтов интересов, связанных с содержанием настоящей статьи.

Участие авторов. Кроме того, все авторы одобрили финальную версию статьи перед публикацией, выразили согласие нести ответственность за все аспекты работы, подразумевающую надлежащее изучение и решение вопросов, связанных с точностью или добросовестностью любой части работы».
